# Multiple polygenic score approach in colorectal cancer risk prediction

**DOI:** 10.1038/s41598-025-21956-w

**Published:** 2025-10-30

**Authors:** Shangqing Joyce Jiang, Minta Thomas, Elisabeth A. Rosenthal, Amanda I. Phipps, Lori C. Sakoda, Franzel J. B. van Duijnhoven, Andrew J. Pellatt, Christy L. Avery, Sonja I. Berndt, D. Timothy Bishop, Sergi Castellví-Bel, Andrew T. Chan, Robert C. Grant, Chris Gignoux, Andrea Gsur, Marc J. Gunter, Christopher A. Haiman, Michael Hoffmeister, Gail P. Jarvik, Mark A. Jenkins, Temitope O. Keku, Sébastien Küry, Jeffrey K. Lee, Loic Le Marchand, Victor Moreno, Polly A. Newcomb, Christina C. Newton, Shuji Ogino, Julie R. Palmer, Rachel Pearlman, Conghui Qu, Robert E. Schoen, Caroline Y. Um, Bethany Van Guelpen, Kala Visvanathan, Veronika Vymetalkova, Emily White, Michael O. Woods, Elizabeth A. Platz, Hermann Brenner, Douglas A. Corley, Iris Landorp Vogelaar, Li Hsu, Ulrike Peters

**Affiliations:** 1 University of Washington, Seattle, WA USA; 2 Public Health Sciences Division, Fred Hutchinson Cancer Center, Seattle, WA USA; 3 Department of Medicine, Division of Medical Genetics, University of Washington, Seattle, WA USA; 4 Department of Epidemiology, University of Washington, Seattle, WA USA; 5 Kaiser Permanente Division of Research, Oakland, CA USA; 6 Department of Health Systems Science, Kaiser Permanente Bernard J. Tyson School of Medicine, Pasadena, CA USA; 7 Division of Human Nutrition and Health, Wageningen University & Research, Wageningen, The Netherlands; 8 Intermountain Health, Salt Lake City, UT USA; 9 Department of Epidemiology, University of North Carolina at Chapel Hill, Chapel Hill, NC USA; 10 Division of Cancer Epidemiology and Genetics, National Cancer Institute, National Institutes of Health, Bethesda, MD USA; 11 Leeds Institute of Cancer and Pathology, University of Leeds, Leeds, UK; 12 Gastroenterology Department, Centro de Investigación Biomédica en Red de Enfermedades Hepáticas y Digestivas (CIBEREHD), Hospital Clínic, Institut d’Investigacions Biomèdiques August Pi i Sunyer (IDIBAPS), University of Barcelona, Barcelona, Spain; 13 Division of Gastroenterology, Massachusetts General Hospital, Harvard Medical School, Boston, MA USA; 14 Channing Division of Network Medicine, Brigham and Women’s Hospital, Harvard Medical School, Boston, MA USA; 15 Clinical and Translational Epidemiology Unit, Massachusetts General Hospital, Harvard Medical School, Boston, MA USA; 16 Broad Institute of Harvard and MIT, Cambridge, MA USA; 17 Department of Epidemiology, Harvard T.H. Chan School of Public Health, Harvard University, Boston, MA USA; 18 Department of Immunology and Infectious Diseases, Harvard T.H. Chan School of Public Health, Harvard University, Boston, MA USA; 19 Princess Margaret Cancer Centre, University Health Network, Toronto, Canada; 20 Colorado Center for Personalized Medicine, University of Colorado - Anschutz Medical Campus, Aurora, CO USA; 21 Center for Cancer Research, Medical University of Vienna, Vienna, Austria; 22 Nutrition and Metabolism Branch, International Agency for Research on Cancer, World Health Organization, Lyon, France; 23 Cancer Epidemiology and Prevention Research Unit, School of Public Health, Imperial College London, London, UK; 24 Center for Genetic Epidemiology, Department of Population and Public Health Sciences, Keck School of Medicine, University of Southern California, Los Angeles, CA USA; 25 Division of Clinical Epidemiology and Aging Research, German Cancer Research Center (DKFZ), Heidelberg, Germany; 26 Department of Genome Sciences, University of Washington School of Medicine, Seattle, WA USA; 27 Centre for Epidemiology and Biostatistics, Melbourne School of Population and Global Health, The University of Melbourne, Melbourne, VIC Australia; 28 Center for Gastrointestinal Biology and Disease, University of North Carolina, Chapel Hill, NC USA; 29 Service de Génétique médicale, Nantes Université, CHU de Nantes, Nantes, F-44000 France; 30 Department of Gastroenterology, Kaiser Permanente San Francisco Medical Center, San Francisco, CA USA; 31 Division of Gastroenterology, University of California, San Francisco, San Francisco, CA USA; 32 University of Hawaii Cancer Center, Honolulu, HI USA; 33 Oncology Data Analytics Program (ODAP), Unit of Biomarkers and Suceptibility (UBS), Catalan Institute of Oncology (ICO), L’Hospitalet del Llobregat, Barcelona, 08908 Spain; 34 ONCOBELL Program, Bellvitge Biomedical Research Institute (IDIBELL), L’Hospitalet de Llobregat, Barcelona, 08908 Spain; 35 Consortium for Biomedical Research in Epidemiology and Public Health (CIBERESP), Madrid, 28029 Spain; 36 Department of Clinical Sciences, Faculty of Medicine and health Sciences, Universitat de Barcelona Institute of Complex Systems (UBICS), University of Barcelona (UB), L’Hospitalet de Llobregat, Barcelona, 08908 Spain; 37 School of Public Health, University of Washington, Seattle, WA USA; 38 Department of Population Science, American Cancer Society, Atlanta, Georgia; 39 Program in MPE Molecular Pathological Epidemiology, Department of Pathology, Brigham and Women’s Hospital, Harvard Medical School, Boston, MA USA; 40 Institute of Science Tokyo, Tokyo, Japan; 41 Slone Epidemiology Center, at Boston University, Boston, MA USA; 42 Division of Human Genetics, Department of Internal Medicine, The Ohio State University Comprehensive Cancer Center, Columbus, OH USA; 43 Departments of Medicine and Epidemiology, University of Pittsburgh Medical Center, Pittsburgh, PA USA; 44 Department of Diagnostics and Intervention, Oncology Unit, Umeå University, Umeå, Sweden; 45 Wallenberg Centre for Molecular Medicine, Umeå University, Umeå, Sweden; 46 Department of Epidemiology, Johns Hopkins Bloomberg School of Public Health, Baltimore, MD USA; 47 Department of Molecular Biology of Cancer, Institute of Experimental Medicine of the Czech Academy of Sciences, Prague, Czech Republic; 48 Institute of Biology and Medical Genetics, First Faculty of Medicine, Charles University, Prague, Czech Republic; 49 Discipline of Genetics, Memorial University of Newfoundland, St. John’s, Canada; 50 Sidney Kimmel Comprehensive Cancer Center at Johns Hopkins, Baltimore, MD USA; 51 German Cancer Consortium (DKTK), German Cancer Research Center (DKFZ), Heidelberg, Germany; 52 Department of Public Health, Erasmus MC, University Medical Center, Rotterdam, The Netherlands; 53 Department of Biostatistics, University of Washington, Seattle, WA USA

**Keywords:** Polygenic risk score, Multi-trait PRS, Colorectal cancer, Cancer, Genetics

## Abstract

Recent studies have demonstrated that for various diseases, incorporating polygenic risk scores (PRSs) for other traits and diseases into the PRS-based risk prediction model may improve predictive performance – known as Multiple Polygenic Score (MPS) approach. We aimed to examine whether the MPS approach improves colorectal cancer (CRC) risk prediction. We included 2,187 non-CRC PRSs from the polygenic Score (PGS) Catalog and used machine learning (ML) models to select the most predictive non-CRC PRSs, utilizing individual-level data from 31,257 CRC cases and 33,408 controls. An independent dataset from the Genetic Epidemiology Research in Adult Health and Aging (GERA) cohort (4,852 cases and 67,939 controls) was randomly split into subsets for model estimation and validation. The model combined MPS with two existing CRC-PRSs based on known loci and genome-wide genotyping. We then assessed model performance by calculating the area under the receiver operating curve (AUC) in the validation set and performed 1,000 bootstrapped iterations to evaluate AUC improvements. The ML model selected 337 non-CRC PRSs predictive of CRC risk. Adding MPS to the CRC-PRSs significantly improved AUC by 0.017 (95% CI: 0.011–0.022, *p* < 0.0001) when combined with known-loci CRC-PRS, 0.005 (95% CI: 0.002–0.007, *p* = 0.0005) with genome-wide CRC-PRS, and 0.004 (95% CI: 0.002–0.006, *p* = 0.0005) with both the known loci and genome-wide CRC-PRSs. These findings demonstrate MPS’s potential to refine CRC risk prediction models and highlight opportunities for further advancements in risk prediction.

**Supplementary Information:**

The online version contains supplementary material available at 10.1038/s41598-025-21956-w.

## Introduction

Colorectal cancer (CRC) is one of the most common causes of cancer death^1^. In 2024, more than 150,000 individuals are projected to be diagnosed in the US, with CRC and more than 50,000 deaths are expected to be attributed to the disease^2^. However, CRC screening can effectively reduce the incidence of CRC, diagnose patients at an earlier stage, remove precursor lesions (polyps and adenomas), and ultimately improve health outcomes^1^. One promising approach to guiding personalized CRC screening is the use of risk prediction models.

Several CRC risk prediction models have been developed, primarily based on environmental and lifestyle risk factors, such as smoking, alcohol, diet, obesity, and diabetes^3–5^. In recent years, prediction scores based on common genetic variants across the human genome have shown promise in identifying individuals with a higher genetic risk of CRC^6,7^. These predictors are known as Polygenic Risk Scores (PRSs). PRSs leverage information based on either known loci from genome-wide association studies (GWAS) or genome-wide risk prediction models including a large number of common variants^8^. PRS tools developed for CRC have been shown to identify individuals at a higher risk for CRC, and adding such PRS into existing clinical risk prediction scores improves risk prediction^6,9^.

There have been many advances in the development of PRSs. With more data available in GWAS, more known loci have been identified and their associations with CRC are becoming better understood^7,10^. In addition, machine learning (ML) models that use genome-wide information have demonstrated further improvements in PRS development^11,12^. In independent validations, these CRC-PRSs developed from genome-wide information using ML models had a high area under the receiver operating curve (AUROC, AUC), a measure for predictive performance, ranging from 0.63 to 0.65, demonstrating their promise in predicting CRC risk using genetic data^6,7^.

Another recent advancement in genetic risk prediction is the Multiple PRS (MPS) approach, which incorporates PRSs for multiple related traits into a single risk prediction model. The motivation of our study is two-fold. First, there are many risk factors for CRC, such as type 2 diabetes, smoking, obesity, and chronic inflammation^13–15^, have genetic components. For example, type 2 diabetes is a known risk factor for CRC, and PRSs developed for diabetes have demonstrated strong predictive performance for type 2 diabetes^16^. Including PRSs for these risk factors may add complementary information beyond CRC-PRSs. Second, traditional penalization models used in developing PRSs, which shrink the effect of less predictive variants, may lose valuable information, that could potentially be captured by PRSs for other traits^8^.

Previous studies have shown that including PRSs from other traits can improve risk prediction for mental health outcomes and diabetes among others^17–19^. However, this approach has not yet been tested for CRC. As the goal is to improve risk prediction, a more comprehensive approach using a broad set of PRSs, including those not directly associated with CRC, is needed to evaluate whether this MPS approach is effective for CRC risk prediction^17,18,20^.

To address this gap, our study aims to assess whether a comprehensive MPS approach can enhance CRC risk prediction compared to a PRS risk prediction model developed solely for CRC = specific PRSs (i.e., CRC-PRS model).

## Methods

**Fig. 1:**
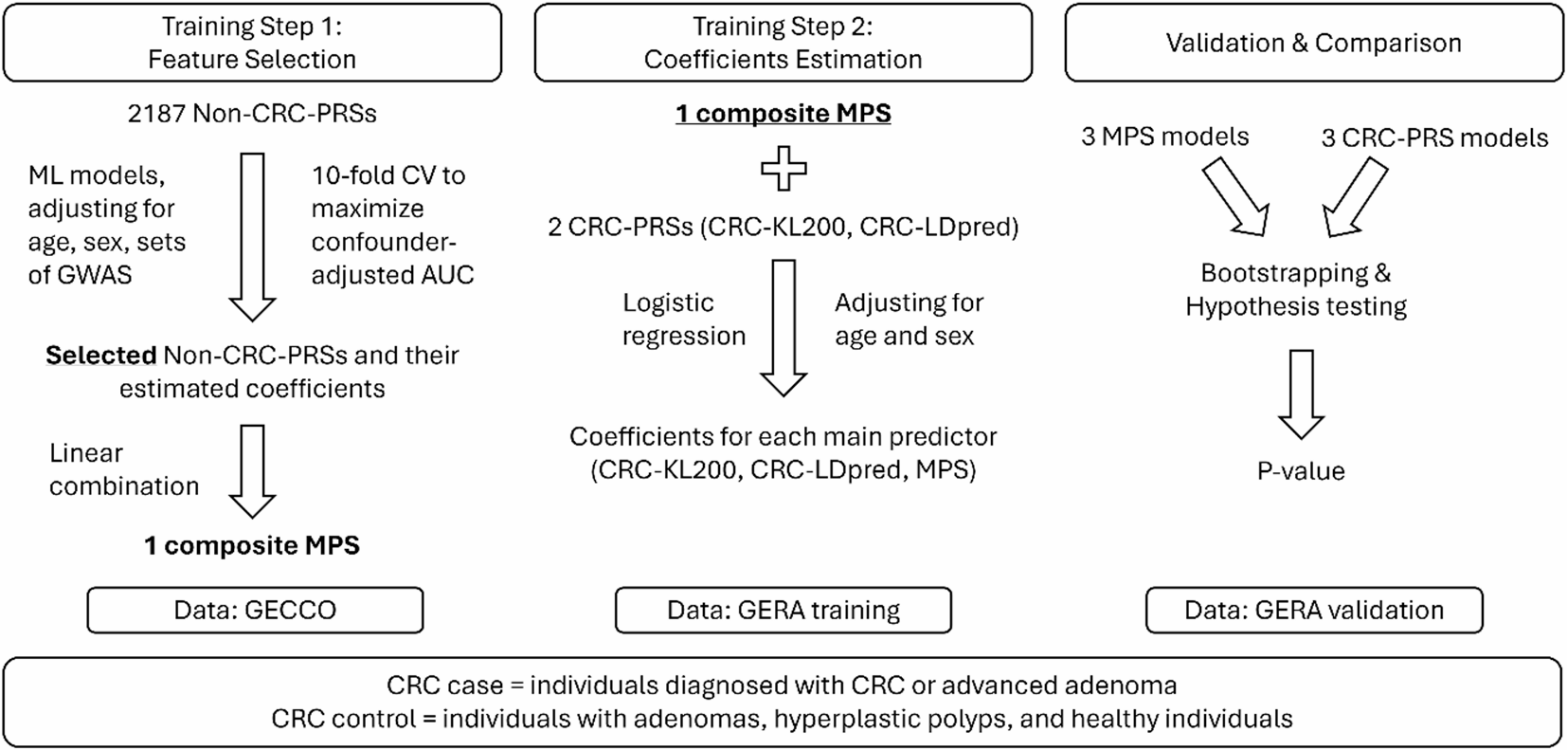
Overall approach: The first step was to select the most predictive non-CRC PRSs among 2,187 non-CRC PRSs using ML models (Lasso, Ridge and Elastic Net) with 10-fold CV. This step was performed using GECCO data. We adjusted for age, sex, and GWAS platforms. We selected the best-performing model with the highest confounder-adjusted AUC. Afterwards, we constructed a composite MPS by taking the linear combination of selected non-CRC PRSs, weighted by their coefficient estimates. The second step was to construct risk prediction models by adding MPS to CRC-PRSs-only models. We adjusted for age and sex in this step. The data were the training subset of GERA cohort. The last step was to validate the confounder-adjusted AUC using the validation dataset. We used bootstrapping to compare confounder-adjusted AUC in CRC-PRS-only models and MPS models and calculated the two-sided p-values.

The overall approach for developing and validating the MPS risk prediction model for CRC is illustrated in Fig. 1. Briefly, our approach involved three steps. The first step was to incorporate all PRSs for other traits (i.e., non-CRC PRSs) available in the polygenic score (PGS) Catalog^16^ into our large GWAS dataset for CRC from the Genetic and Epidemiology of CRC Consortium (GECCO) and Colon Cancer Family Registry (CCFR). Detailed information on the imputation process, GWAS, and sub-studies can be found in our previous publications^6,10^. This dataset included 64,665 individuals of European ancestry, comprising 31,257 cases diagnosed with CRC or advanced adenomas (AA), and 33,408 controls. The mean age of participants was 64.8 years (SD = 11.4) and 50.2% were females (Table 1). To select the most predictive PRSs, we used ML models including Lasso, Ridge, and Elastic Net. The best performing model based on cross-validation (CV) performance returned a subset of selected non-CRC PRSs along with their estimated coefficients.

**Table 1:** Characteristics of the datasets: GECCO and CCFR and GERA.

GECCO and CCFR					
	Cases (*N* = 31,257)			Controls (*N* = 33,408)	Overall (*N* = 64,665)
	CRC (*N* = 26,884)	Advanced adenoma (*N* = 4,373)	Total (*N* = 31,257)	Total	Total
Age, mean (SD)	65.7 (11.4)	64.3 (8.0)	65.5 (11.0)	64.2 (11.6)	64.8 (11.4)
Female, n (%)	12,811 (47.7%)	1,858 (42.5%)	14,669 (46.9%)	17,780 (53.2%)	32,449 (50.2%)

GERA					
	Cases (*N* = 4,852)			Controls (*N* = 67,939)	Overall (*N* = 72,791)
	CRC (*N* = 1,311)	Advanced adenoma (*N* = 3,541)	Total (*N* = 4,852)	Total	Total
Age, mean (SD)	70.9 (11.7)	68.5 (9.1)	69.2 (9.9)	71.3 (13.3)	71.1 (13.1)
Female, n (%)	674 (51.4%)	1,643 (46.4%)	2,317 (47.8%)	40,203 (59.2%)	42,520 (58.4%)

Abbreviations. CRC: colorectal cancer. GECCO: Genetic and Epidemiology of CRC Consortium. CCFR: Colon Cancer Family Registry. GERA: Genetic Epidemiology Research in Adult Health and Aging (GERA) cohort. SD: standard deviation.

The second step was to generate a CRC risk prediction model using logistic regression, which include both CRC and selected non-CRC PRSs. The third step was to validate the performance of the proposed model. For steps two and three, we randomly split the Genetic Epidemiology Research in Adult Health and Aging (GERA) cohort into subsets. The GERA cohort is a large-scale, community-based research project studying genetic factors related to age-related diseases in a demographically diverse group of adults. It integrates extensive longitudinal medical records with genetic information from Kaiser Permanente, Northern California, a multi-center integrated healthcare delivery system^21^. Among the 72,791 individuals, there were 4,852 cases and 67,939 controls. The mean age of participants was 71.1 years (SD = 13.1) and 58.4% were females (Table 1). Detailed descriptions of this dataset have been published previously^6^. All study protocols were approved by Fred Hutchinson Institutional Review Board (IRB) and Kaiser Permanente Northern California IRB, and informed consent was obtained from all participants in accordance with the Helsinki Accord.

The following sections provide a detailed description of each step.

### CRC-PRSs

We used two CRC-PRSs, both of which we published previously. Briefly, the first CRC-PRS was based on 204 GWAS known loci (CRC-KL200); and the second one was based on genome-wide approach constructed using LDpred2 (CRC-LDpred). For more details of these two CRC-PRSs, please refer to previous publications^7,22^.

### Non-CRC PRSs

**Fig. 2:**
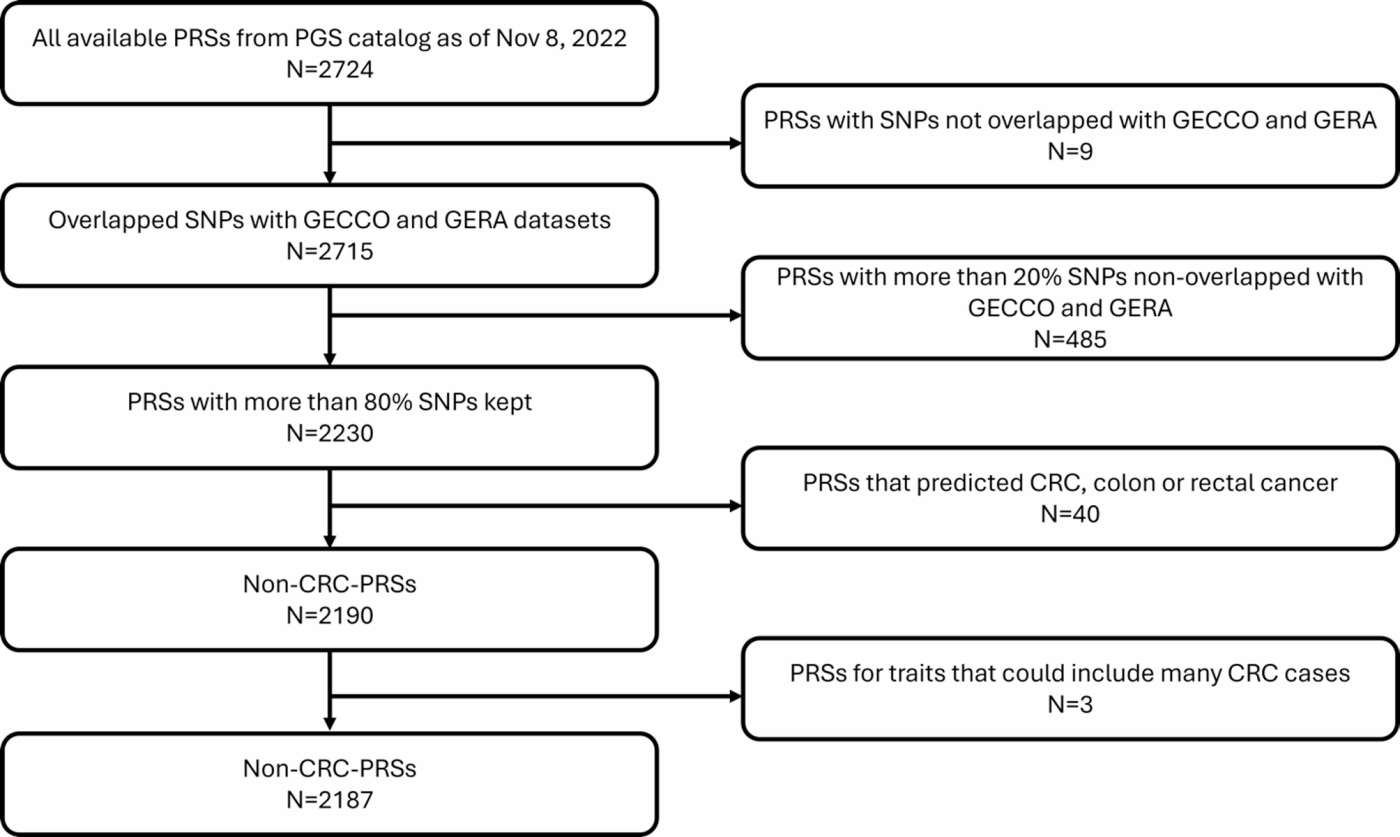
Selection of non-CRC-PRSs: We downloaded all PRSs available as of Nov 8, 2022, from PGS Catalog. We first excluded 9 PRSs without overlapping SNPs with GECCO, CCFR, and GERA datasets. Then, we further excluded 485 PRSs with at least 20% SNPs unavailable in GECCO, and GERA datasets. Because our focus was non-CRC PRSs, we excluded 40 PRSs that predicted CRC, colon cancer, and rectal cancer. Additionally, three PRSs were excluded because they predicted the trait where many CRC cases were included. Eventually, we had 2,187 non-CRC PRSs.

To examine whether incorporating non-CRC PRSs could enhance predictive performance, we comprehensively incorporated all available non-CRC PRSs without prior selection. We downloaded scoring files for all 2,724 PRSs’ from the PGS Catalog as of November 8, 2022^16^. The vast majority of SNPs included in the non-CRC PRSs had minimal missingness, with a median of 2% missingness. PRSs with more than 20% missing SNPs were excluded to avoid substantial loss of predictive accuracy (Table S1). Additionally, we excluded PRSs specifically designed to predict CRC, colon cancer, and rectal cancer. To minimize overlap with CRC cases, we also excluded three PRSs related to two broader traits—gastrointestinal cancer and rectal/anal cancer. After these exclusions, 2,187 non-CRC PRSs remained for analysis in the ML model (Fig. 2).

Among these 2,187 non-CRC PRSs, some were associated with precursor lesions of CRC, including rectal polyps, benign neoplasms of the colon, and benign neoplasms of the digestive system. These PRSs were retained in the analysis, as they predict precursors of CRC rather than CRC itself.

### Statistical analysis

#### PRS calculation

We calculated all PRSs for each individual using the genotype data of GECCO and CCFR for step one and of GERA for steps two and three. For each PRS, we calculated the weighted sum of effect alleles across all common variants included in that specific PRS. Scoring files for each PRS were obtained from the PGS Catalog, which provided information on SNPs, effect alleles, reference alleles, and corresponding effect weights^16^. All PRSs were standardized to have a mean of zero and a standard deviation (SD) of one, based on the overall sample mean.

#### Model Building and validation

Step 1. Selection of non-CRC PRSs.

The first step involved identifying the most predictive non-CRC PRSs for CRC risk. We developed ML models, including Ridge, Lasso, and Elastic Net models on non-CRC PRSs derived from the GECCO and CCFR datasets. To determine the optimal penalization tuning parameter, we performed 10-fold CV, selecting the value that maximized the confounder-adjusted AUC. This metric standardizes confounders distribution in the case group to match that of the control group, ensuring that the predictive performance is attributed solely to primary predictors of interest.

Each ML model was adjusted for age, sex, and genotyping platform, with penalization applied only to PRSs, while age, sex, and genotyping platform remained unpenalized. After performing 10-fold CV, we calculated the mean confounder-adjusted AUC and selected the model with the highest value. Using the optimal tuning parameters, we re-fitted the model on the full GECCO and CCFR dataset to estimate the coefficients for non-CRC PRSs. At the end of this step, we generated an MPS score using best performing ML model. This score represents a linear combination of selected non-CRC PRSs weighted by the estimated coefficients.

Step 2. Developing risk prediction models.

The second step was to construct risk prediction models including both CRC-PRS and MPS. We considered six models in total: three CRC-PRS models (CRC-KL200 only, CRC-LDpred only, and a combination of CRC-KL200 and CRC-LDpred), and three MPS + CRC-PRS models where MPS was added to each of the three CRC-PRSs models. Since we had significantly reduced the number of predictors using ML methods and had a sufficiently large sample size for model development, we applied logistic regression model to combing PRSs. CRC-PRSs and MPS were normalized to have a mean of zero and a standard deviation (SD) of one, and all models were adjusted for age, and sex. We used the GERA dataset for this step, where 72,791 adults of European ancestry based on genetic data were included.

We randomly split GERA into two equally sample sized. We used one set for estimating the parameters for each of the six models (step 2), and the second set for validation (see step 3. Validation and Comparison). We developed risk prediction models for combined and sex-stratified analysis (i.e., separate models for males and for females). Our primary outcome was advanced neoplasia, including CRC or advanced adenomas, and with all other individuals classified as controls. As a secondary analysis, we examined the CRC as outcome and defining controls as all individuals, except those with CRC or advanced adenoma.

Step 3. Validation and Comparison.

We evaluated the performance of the six risk prediction models using the validation subset of the GERA data sets. For each model, we estimated the confounder-adjusted AUC, accounting for age and sex, and obtained the standard errors (SE) and the 95% confidence intervals (CI) based on 1000 bootstrap samples.

We conducted three pairs of comparisons: (1) combined CRC-KL200 and MPS vs. CRC-KL200, (2) combined CRC-LDpred and MPS vs. CRC-LDpred only, and (3) combined CRC-KL200, CRC-LDpred and MPS vs. CRC-KL200 and CRC-LDpred. To evaluate the improvement in model performances, we first calculated the difference in confounder-adjusted AUC by subtracting the AUC of each CRC-PRS model from the corresponding MPS and CRC-PRS model in the GERA validation dataset (for example: CRC-KL200 + MPS minus CRC-KL200), We then repeated this process across 1,000 bootstrapped samples to estimate the SE of the AUC difference, and derive the 95% CI.

To determine statistical significance, we computed the z-score for the AUC difference and obtained two-sided p-values. A p-value below 0.05 was considered statistically significant.

## Results

Step 1. Model selection of non-CRC-PRSs.

The best performing ML model was an Elastic Net model. In 10-fold CV, the model maximized the AUC when the alpha value was around 0.75 and the lambda value was around 0.003. This model achieved a mean confounder-adjusted AUC of 0.607 across 10-fold CV in GECCO data (Figure S1) and identified 337 non-CRC PRSs with non-zero coefficient estimates. The list of selected 337 non-CRC PRSs along with their coefficient estimates can be found in Table S2. Non-CRC PRSs with the highest absolute coefficient values were those for benign neoplasm of colon, any cancer, college education, hemorrhoids, and body mass index (BMI).

**Table 2:** Main analysis: odds ratio (OR) estimates, 95% CI and p-values for each predictor in logistic regression model.

Model No.	Predictors	OR estimates	95% CI: Lower limit	95% CI: Upper limit	*P*-value
1	CRC-KL200	1.49	1.43	1.55	< 0.0001
2	CRC-LDpred	1.63	1.57	1.72	< 0.0001
3	CRC-KL200	1.15	1.08	1.21	< 0.0001
	CRC-LDpred	1.51	1.42	1.58	< 0.0001
4	Composite MPS	1.22	1.16	1.28	< 0.0001
	CRC-KL200	1.36	1.30	1.42	< 0.0001
5	Composite MPS	1.13	1.07	1.19	< 0.0001
	CRC-LDpred	1.54	1.46	1.62	< 0.0001
6	Composite MPS	1.11	1.05	1.16	< 0.0001
	CRC-KL200	1.12	1.05	1.19	0.0001
	CRC-LDpred	1.45	1.36	1.54	< 0.0001

Abbreviations. CRC: colorectal cancer. CI: confidence interval. MPS: multiple PRS.

Step 2. Developing risk prediction models.

First, we evaluated the performance of all models on datasets where cases included advanced neoplasia, including CRC and advanced adenomas, with all other individuals classified as controls. Across all six models, both CRC-related PRSs and MPS were statistically significant (Table 2). For example, in Model 6, the odds ratio (OR) with 95% confidence interval (CI) for MPS, CRC-KL200, and CRC-LDpred were 1.11 (95% CI 1.05–1.16 with p-value < 0.0001), 1.12 (95% CI 1.05–1.19 with p-value 0.0001), and 1.45 (95% CI 1.36–1.54 with p-value < 0.0001), respectively (Table 2). The results of the sex-stratified analysis were given in Table S3, and Table S4. In the risk prediction models for females, most predictors had statistically significant ORs. However, CRC-KL200 became non-significant when CRC-LDpred was also included as a predictor (Table S3). All CRC-PRS and MPS + CRC-PRS remained significant in all models for males. However, unlike in models for females, CRC-KL200 remained significant even when CRC-LDpred was included in the model (CRC-KL200 in Model 3: OR 1.19 (95% CI 1.09–1.28 with p-value < 0.0001); in Model 6: OR 1.16 (95% CI 1.07–1.25 with p-value 0.0003). MPS continued to be statistically significant even when both CRC-KL200 and CRC-LDpred were included (MPS in Model 6: OR 1.12 (95% CI 1.05–1.20 with p-value 0.001) (Table S4). We also assessed the performance of the model when cases were restricted to individuals with CRC only. MPS and CRC-KL200 become non-significant predictors when the model included CRC-LDpred, and the results were included in Table S5.

Step 3. Validation and Comparison.

AUC analysis for advanced neoplasia.

**Table 3:** Main analyses: AUC (95% CI) estimates of risk prediction models and point estimate of differences in AUCs between models with and without MPS.

Main analysis			
Model No	Main predictors	AUC (95% CI)	Point estimate of difference in AUC (95% CI) and P-value
1	CRC-KL200	0.600 (0.589–0.612)	
2	CRC-LDpred	0.631 (0.620–0.643)	
3	CRC-KL200, CRC-LDpred	0.632 (0.621–0.644)	
4	CRC-KL200, MPS	0.617 (0.606–0.629)	0.017^#^ (0.011–0.022) pval < 0.0001
5	CRC-LDpred, MPS	0.636 (0.625–0.648)	0.005^*^ (0.002–0.007) pval 0.0005
6	CRC-KL200, CRC-LDpred, MPS	0.636 (0.625–0.648)	0.004^^^ (0.002–0.006) pval 0.0005

Abbreviations. AUC: area under the Receiver Operating Curve. CRC: colorectal cancer. CI: confidence interval. MPS: multiple PRS. ^#^ Model 4 AUC – Model 1 AUC; ^*^Model 5 AUC-Model2 AUC; and ^^^ Model 6 AUC – Model 3 AUC.

When comparing individuals with advanced neoplasia to all others, the AUC was 0.600 (95%CI: 0.589–0.612), 0.631 (95%CI: 0.620–0.643), and 0.632 (95%CI: 0.621–0.644) for CRC-KL200, CRC-LDpred, and CRC-KL200 + CRC-LDpred, respectively (Table 3). The AUCs for the three MPS + CRC-PRS models are 0.617 (95%CI: 0.606–0.629), 0.636 (95%CI: 0.625–0.648), and 0.636 (95%CI: 0.625–0.648), respectively (Table 3).

Incorporating MPS into the known loci model increased the AUC 0.017 (95% CI: 0.011–0.022; *p* < 0.0001); the CRC-LDpred model 0.005 (95%CI: 0.002–0.007; *p* = 0.0005); and both the CRC-KL200 and CRC-LDpred model 0.0004 (95%CI: 0.002–0.006; *p* = 0.0005) (Table 3, Figure S2-S4).

The results of the sex-stratified analysis were given in Table S6 and Table S7. For females, incorporating MPS into the known loci model increased the AUC 0.012 (95% CI: 0.003–0.021); the CRC-LDpred model 0.004 (95%CI: 0.001–0.009); and both the CRC-KL200 and CRC-LDpred model 0.003 (95%CI: 0.0-, 0.007) (Table S6), whereas for males, incorporating MPS into the known loci model increased the AUC 0.016 (95% CI: 0.009–0.023); the CRC-LDpred model 0.005 (95%CI: 0.001–0.010); and both the CRC-KL200 and CRC-LDpred model 0.004 (95%CI: 0.001–0.008) (Table S7),

CRC analysis.

When we restricted the analysis to CRC only (excluding advanced adenoma) the addition of MPS improved the risk prediction performance of the CRC-KL200 model (AUC difference = 0.015; 95%CI: 0.007, 0.023; *p* = 0.0002), but did not improve the performance significantly when CRC-LDpred was the main predictor (AUC difference = 0.002; 95%CI: 0.000, 0.015; *p* = 0.13) or when CRC-LDpred and CRC-KL200 were both included (AUC difference = 0.002; 95%CI: 0.000, 0.004; *p* = 0.098) (Table S8).

## Discussion

We found that MPS was an independent predictor beyond CRC-PRSs in both the combined and sex-stratified analysis. This suggests that the information captured by non-CRC PRSs contributed to advanced colorectal neoplasia prediction. However, when CRC-LDpred was included in the model, the OR estimate of MPS was rather small. This explained why MPS did not noticeably improve the AUC, especially when CRC-LDpred was included in the model.

Among the 337 non-CRC PRSs selected by the Elastic Net model, those with the largest OR estimates were for predicting benign neoplasm, precursor lesions of CRC. This may explain why in the analysis focusing on CRC, excluding advanced adenomas, MPS’s contribution in risk prediction was somewhat weakened. Together, these findings indicate that the added value of MPS may primarily reflect risk for precursor lesions rather than invasive CRC, underscoring the importance of evaluating both endpoints in risk prediction.

Although MPS improved the AUC significantly, the increment in AUC was small. Consistent with our findings, Truong et al. found that incorporating more than 2000 PRSs to predict coronary artery disease, the increase in AUC ranged from 0.003 to 0.023 compared to a model without MPS, and the p-value for the increase in AUC were all highly significant (*p* < 2e-16)^19^. Additionally, Krapohl et al. found that the MPS approach improved the variance explained of educational achievement, general cognitive ability, and BMI by 0.011 to 0.016 and this increase was statistically significant (*p* < 0.004)^17^. The magnitude of the effect size of MPS in these findings is in line with ours^17,19^.

There are at least two factors that may influence whether an MPS approach can contribute to risk prediction. The first one is the genetic architecture of a given trait, and the second factor is how much heritability has already been captured by the trait/disease-specific PRS (i.e., CRC-PRSs in our study). CRC is a complex trait, and both genetic variants and environmental risk factors contribute to its risk deposition^23^. CRC is highly polygenic and impacted by both common genetic risk factors as well as rare variants in high-penetrance genes, such as mismatch repair genes^23^. Rare variants in high-penetrance genes can increase the risk of CRC to up by 70%^23^, and account for 3–5% of CRC cases^24,25^. To date, more than 200 common variants have been discovered in GWAS of CRC, which explain close to 20% of the familial risk; however, it is estimated that all common variants (including undiscovered) explain over 70% of the familial risk^7,22^. This suggests that in addition to the known GWAS loci, other common variants can contribute to the risk prediction. This is supported by our finding that the AUC was improved from 0.012 to 0.017 after we added MPS to CRC-KL200.

However, because CRC-LDpred was developed using genome-wide data from a large sample of individuals with European ancestry and Asian ancestry, and constructed by LDpred2, one of the state-of-the-art methods for developing PRS^11,26^, it is possible that CRC-LDpred has captured the majority of the genome-wide information of CRC, especially undiscovered common variants. As a result, the incremental improvement from incorporating MPS may be limited. Instead, integrating non-genetic risk factors, such as lifestyle, comorbidities, and environmental exposures, has been shown to significantly improve CRC risk prediction when combining with CRC PRS^9,26,27^. Additionally, new methods for PRS development, such as incorporating functional information, may also help improve risk predictive performance^28–30^. Future studies should focus on developing more effective approaches to improve PRS risk prediction.

There are two potential mechanisms by which the MPS approach might improve risk prediction. The first one is pleiotropy, i.e., the genetic correlations among complex traits^31^. Recent pleiotropic analyses have found novel genetic risk factors associated with CRC^32–34^. These novel genetic risk factors might not have been included in the CRC-PRSs but may be captured by PRS for other traits. Secondly, methods of PRS development may over-shrink the effect sizes of common variants, leading to a loss of information of CRC-PRSs^35–37^. In this case, these common variants can be supplemented by PRSs of other traits in the MPS approach.

Overall, our study shows that combining multiple PRSs yields modest but statistically significant improvements in CRC prediction. The small increments are consistent with prior studies in CRC and other complex traits^17,19^. While these gains in AUC may have limited direct clinical impact, they represent refinements in risk modeling. Multi-PRS models capture complementary information and integrating them with established risk factors such as BMI, smoking, or diabetes may further improve risk prediction. Given the moderate predictiveness of genetic risk prediction models, the greatest utility of PRSs - including MPS - may lie in risk stratification: identifying individuals at elevated risk who might benefit from earlier or more frequent screening, or in enhancing the performance of existing screening tools such as fecal blood tests or blood-based biomarkers.

An important strength of our study is that we designed the MPS approach in a comprehensive way. First, instead of pre-selecting traits based on the current understanding of CRC risk factors, we considered all available non-CRC PRSs from the PGS Catalog^16^. Then, we used ML models (ridge, lasso, and elastic net) to identify those predictive of CRC. This ensured an unbiased model and allowed us to incorporate a broad set of 337 non-CRC PRSs while addressing collinearity through regularized regression. Second, all models were validated in a separate dataset, ensuring that AUC estimates reflect independent evaluation and avoiding overfitting. Third, we used confounder-adjusted AUC^38^, which measures the predictive performance of CRC-PRSs and MPS only, excluding prediction attributable to factors known to predict CRC, such as age and sex. Using this metric, we were able to make fair comparison between CRC-PRS models and MPS models.

However, limitations are also noteworthy. First, our study only focused on individuals of European ancestry, as most PRSs were developed for this population^16^. Extending these analyses to diverse ancestral groups will be important to assess generalizability. Second, we examined the linear relationship between CRC and PRSs. A previous study has used deep neural networks, which captures non-linearity, and found that it performed equally well or outperformed ridge regression^39^. However, a recent article found that XGBoost, another ML method that captures non-linearity, improved the predictive performance of MPS models compared to lasso, but the improvement was only via covariates rather than PRSs^40^. Future studies should examine how and why different ML models perform in the context of MPS in CRC and other diseases.

## Conclusion

In conclusion, combining multiple PRSs yields modest but statistically significant improvements in CRC risk prediction. Although the absolute gains are limited, these refinements may still support risk stratification for individuals at elevated risk, potentially guiding earlier or more frequent screening and integration with other screening tools. Future work should focus on incorporating lifestyle and clinical factors, leveraging functional annotation, and validating models across diverse populations to advance personalized CRC prevention and early detection.

## Supplementary Information

Below is the link to the electronic supplementary material.

Supplementary Material 1

Supplementary Material 2

## Data Availability

GECCO have been deposited in the database of Genotypes and Phenotypes (dbGaP) under accession numbers [phs001078.v1.p1] (https://www.ncbi.nlm.nih.gov/projects/gap/cgi-bin/study.cgi?study_id=phs001078.v1.p1), [phs001415.v1.p1] (https://www.ncbi.nlm.nih.gov/projects/gap/cgi-bin/study.cgi?study_id=phs001415.v1.p1), and [phs001315.v1.p1] (https://www.ncbi.nlm.nih.gov/projects/gap/cgi-bin/study.cgi?study_id=phs001315.v1.p1). Genotype data of GERA participants who consented to having their data shared with dbGaP are available from dbGaP under accession [phs000674.v2.p2] (https://www.ncbi.nlm.nih.gov/projects/gap/cgi-bin/study.cgi?study_id=phs000674.v2.p2). The complete GERA data are available upon successful application to the KP Research Bank.
