# Supplementary material for: Multiple polygenic score approach in colorectal cancer risk prediction
Source: Sci Rep. 2025 Oct 30;15:38006. doi: 10.1038/s41598-025-21956-w (PMC12575652; doi:10.1038/s41598-025-21956-w)
Supplement: Supplementary file 2 — Supplementary Material 2 [file 41598_2025_21956_MOESM2_ESM.docx]

**Multiple Polygenic Score Approach in Colorectal Cancer Risk Prediction**

**Supplemental Tables and Figures:** Contents

[Table S2. Selected 337 non-CRC PRSs by Elastic Net model 2](#_Toc166572670)

[Table S3. Stratified analysis: estimated coefficients in logistic regression, female only 16](#_Toc166572671)

[Table S4. Stratified analysis: estimated coefficients in logistic regression, male only 17](#_Toc166572672)

[Table S5. Subgroup analysis: estimated coefficients in logistic regression 18](#_Toc166572673)

[Table S6. Stratified analysis: Comparison of AUCs, females 19](#_Toc166572674)

[Table S7. Stratified analysis: Comparison of AUCs, males 20](#_Toc166572675)

[Table S8. Subgroup analysis: Comparison of AUCs 21](#_Toc166572676)

[Figure S1. Confounder-adjusted AUC in 10-fold CV 22](#_Toc166572677)

[Figure S2. Main analysis: Confounder-adjusted AUC comparing MPS+CRC-KL200 to CRC-KL200 only 23](#_Toc166572678)

[Figure S3. Main analysis: Confounder-adjusted AUC comparing MPS+CRC-LDpred to CRC-LDpred only 24](#_Toc166572679)

[Figure S4. Main analysis: Confounder-adjusted AUC comparing MPS+CRC-KL200+CRC-LDpred to CRC-KL200+CRC-LDpred 25](#_Toc166572680)

# Table S2. Selected 337 non-CRC PRSs by Elastic Net model

| **Polygenic Score ID & Name** | **Reported Trait** | **Estimated Coefficient** |
| --- | --- | --- |
| PGS002019(portability-ldpred2_208) | Benign neoplasm of colon | 0.061474 |
| PGS001811(portability-PLR_208) | Benign neoplasm of colon | 0.013656 |
| PGS000354(PRSWEB_PHECODE10001_UKBB-SAIGE-HRC-X10001_PRS-CS_MGI_20200608) | Any Cancer | 0.011092 |
| PGS002319(cov_EDU_COLLEGE.BOLT-LMM) | College education | -0.009830 |
| PGS002058(portability-ldpred2_455) | Hemorrhoids | -0.006804 |
| PGS000910(PRS_BMI) | Body mass index | 0.006350 |
| PGS001057(GBE_INI1458) | Cereal consumption | -0.005968 |
| PGS001158(GBE_INI23118) | Left leg mass (predicted) | 0.005534 |
| PGS002070(portability-ldpred2_565.1) | Anal and rectal polyp | 0.005507 |
| PGS000938(GBE_HC990) | Varicose veins of lower extremities (time-to-event) | 0.005372 |
| PGS000877(uwGRS53_IR) | Insulin resistance | 0.004865 |
| PGS002148(portability-ldpred2_income) | Average total household income before tax | -0.004645 |
| PGS002096(portability-ldpred2_785) | Abdominal pain | -0.004146 |
| PGS001284(GBE_BIN_FC10006152) | Allergic disease (hay fever, allergic rhinitis, or eczema) | -0.004138 |
| PGS002025(portability-ldpred2_250.1) | Type 1 diabetes | 0.004060 |
| PGS000982(GBE_BIN_FC10002267) | Use of sun / ultraviolet protection (never / rarely) | 0.004047 |
| PGS001763(GBE_INI25562) | WA MO in tract posterior thalamic radiation (R) | -0.004043 |
| PGS000253(IL-6RA) | Interleukin-6 receptor subunit alpha (IL-6RA) serum levels | -0.003942 |
| PGS002356(body_WHRadjBMIz.BOLT-LMM) | Waist-Hip Ratio | 0.003908 |
| PGS001641(GBE_INI25007) | Volume of white matter (normalised for head size) | 0.003882 |
| PGS000356(PRSWEB_PHECODE10001_UKBB-SAIGE-HRC-X10001_LASSOSUM_MGI_20200608) | Any Cancer | 0.003748 |
| PGS000725(PRS_Pancreas) | Pancreatic cancer | 0.003682 |
| PGS001322(GBE_HC276) | Glaucoma | -0.003596 |
| PGS001831(portability-PLR_335) | Multiple sclerosis | 0.003525 |
| PGS000491(PRSWEB_PHECODE174.1_Onco-iCOGS-ER-negative-BRCA_PRS-CS_MGI_20200608) | Breast cancer (female) | 0.003487 |
| PGS000701(snpnet.Urea) | Urea [mmol/L] | -0.003475 |
| PGS002296(PRS2166_HT) | Hypertension | -0.003472 |
| PGS000855(T2D_Lipodystrophy) | Type 2 diabetes (based on SNPs associated with lipodystrophy) | 0.003460 |
| PGS000155(cGRS_Glioma) | Glioma | -0.003455 |
| PGS000265(MMP-10) | Matrix metalloproteinase-10 (MMP-10) serum levels | 0.003430 |
| PGS000803(wGRS41_SLE) | Systemic lupus erythematosus | -0.003384 |
| PGS000798(157SNP_GRS) | Coronary heart disease | -0.003327 |
| PGS000359(PRSWEB_PHECODE145.2_C3-TONGUENAS_PRS-CS_MGI_20200608) | Cancer of tongue | 0.003250 |
| PGS000325(GRS-JIA-Oli-20) | Oligoarthritis Juvenile Idiophatic Arthritis | -0.003249 |
| PGS001088(GBE_INI1588) | Average weekly alcohol consumption (beer and cider) | 0.003237 |
| PGS002240(prscs_prostatecancer) | Prostate cancer | 0.003132 |
| PGS001859(portability-PLR_565.1) | Anal and rectal polyp | 0.003114 |
| PGS000760(VIT) | Vitiligo | -0.003107 |
| PGS001018(GBE_BIN_FC10006160) | Attending social / leisure activities (attend any of the followings in once a week or more often: sports club, gym, pub, social club, religious group, adult edication class, or other group activity) | -0.003034 |
| PGS001470(GBE_INI25352) | Mean ICVF in medial lemniscus on FA skeleton (R) | -0.003023 |
| PGS001574(GBE_INI25827) | Volume of grey matter in Lateral Occipital Cortex, inferior division (R) | -0.002999 |
| PGS001970(portability-PLR_log_platelet_crit) | Platelet crit | -0.002997 |
| PGS002092(portability-ldpred2_728.71) | Contracture of palmar fascia [Dupuytren's disease] | 0.002993 |
| PGS002034(portability-ldpred2_286.12) | Congenital deficiency of other clotting factors (including factor VII) | -0.002991 |
| PGS000320(PRS_BMI) | Body mass index | 0.002900 |
| PGS002804(GIANT_HEIGHT_YENGO_2022_PGS_WEIGHTS_EUR) | Height | 0.002869 |
| PGS002068(portability-ldpred2_562.1) | Diverticulosis | -0.002858 |
| PGS002095(portability-ldpred2_743.1) | Osteoporosis | 0.002818 |
| PGS001386(GBE_INI21049) | Degree bothered by pain in arms/legs/joints in the past 3 months | -0.002808 |
| PGS001985(portability-PLR_logMAR) | logMAR in round (left/right) | 0.002739 |
| PGS002153(portability-ldpred2_less_happy_with_health) | General happiness with own health | -0.002635 |
| PGS000851(T2D_Insulin_Action_Secretion) | Type 2 diabetes (based on SNPs associated with insulin action/secretion) | 0.002584 |
| PGS002358(disease_ASTHMA_DIAGNOSED.BOLT-LMM-BBJ) | Asthma | 0.002581 |
| PGS001373(GBE_INI2217) | Age started wearing glasses or contact lenses | 0.002530 |
| PGS001319(GBE_HC708) | Other metabolic disorders (time-to-event) | -0.002507 |
| PGS001281(GBE_HC86) | Migraine | -0.002458 |
| PGS002159(portability-ldpred2_log_AST) | Aspartate aminotransferase | -0.002442 |
| PGS001548(GBE_INI25864) | Volume of grey matter in Central Opercular Cortex (L) | 0.002428 |
| PGS002365(biochemistry_Glucose.BOLT-LMM-BBJ) | Glucose | 0.002416 |
| PGS002290(GRS10_PUA) | Uric acid level | 0.002398 |
| PGS000221(CCL3) | C-C motif chemokine 3 (CCL3) serum levels | 0.002390 |
| PGS001394(GBE_INI20414) | Freq. of drinking alcohol | 0.002387 |
| PGS001698(GBE_INI25725) | WA ISOVF in tract superior longitudinal fasciculus (L) | 0.002381 |
| PGS002201(portability-ldpred2_log_waist_circ) | Waist circumference | 0.002362 |
| PGS001853(portability-PLR_540) | Appendiceal conditions | -0.002341 |
| PGS000991(GBE_BIN_FC4006144) | Never eat sugar | -0.002320 |
| PGS000620(PRSWEB_PHECODE191.11_C71_LASSOSUM_MGI_20200608) | Cancer of brain | -0.002289 |
| PGS000017(GPS_IBD) | Inflammatory bowel disease | -0.002289 |
| PGS002055(portability-ldpred2_443.9) | Peripheral vascular disease, unspecified | 0.002256 |
| PGS002259(metaPRS_Stroke) | Stroke | -0.002251 |
| PGS001783(1kgeur_gbmi_COPD_pst_eff_a1_b0.5_phiauto) | Chronic obstructive pulmonary disease | -0.002223 |
| PGS001082(GBE_INI23028) | MC VP1 antigen for Merkel Cell Polyomavirus | 0.002207 |
| PGS002226(portability-ldpred2_sodium_urine) | Sodium in urine | 0.002204 |
| PGS002223(portability-ldpred2_sensitive_stomach) | Sensitive stomach | -0.002187 |
| PGS001430(GBE_INI25075) | Mean FA in posterior limb of internal capsule on FA skeleton (L) | 0.002171 |
| PGS000210(LF279) | Lung function (FEV1/FVC) | -0.002137 |
| PGS000011(GRS50) | Coronary artery disease | -0.002091 |
| PGS001893(portability-PLR_calcium) | Calcium | 0.002066 |
| PGS000386(PRSWEB_PHECODE157_GWAS-Catalog-r2019-05-03-X157_PT_UKB_20200608) | Pancreatic cancer | 0.002054 |
| PGS000909(PRS_Headaches) | Headache | -0.002035 |
| PGS002255(PRS_measured) | Physical activity (measured) | -0.002023 |
| PGS000963(GBE_HC1184) | Follicular cysts of skin and subcutaneous tissue (time-to-event) | 0.002017 |
| PGS000332(PRS_BC) | Breast cancer | 0.002005 |
| PGS002012(portability-PLR_years_of_edu) | Qualifications (years of education) | -0.001996 |
| PGS000691(snpnet.Non_albumin_protein) | Non-albumin protein [g/L] | -0.001994 |
| PGS002152(portability-ldpred2_less_alcohol) | Alcohol intake frequency | 0.001986 |
| PGS000853(T2D_Insulin_Secretion_2) | Type 2 diabetes (based on SNPs associated with insulin secretion) | -0.001981 |
| PGS000766(PRS56_CM) | Cutaneous melanoma | 0.001974 |
| PGS002126(portability-ldpred2_ever_smoked) | Ever smoked | -0.001872 |
| PGS001975(portability-PLR_log_pulse_rate) | Pulse rate, automated reading | 0.001844 |
| PGS000206(RISK_PC_FT12) | Risk-taking tendency (4-domain principal component model) | -0.001839 |
| PGS002036(portability-ldpred2_296.2) | Depression | -0.001821 |
| PGS001119(GBE_INI46) | Left hand grip strength | -0.001796 |
| PGS002304(PRS6_FL) | Follicular lymphoma | 0.001795 |
| PGS001689(GBE_INI25705) | WA ISOVF in tract acoustic radiation (R) | 0.001789 |
| PGS000727(AF_PGS) | Atrial fibrillation | 0.001781 |
| PGS002062(portability-ldpred2_496) | Chronic airway obstruction | -0.001780 |
| PGS002166(portability-ldpred2_log_ECG_QRS_duration) | QRS duration | 0.001760 |
| PGS001306(GBE_HC201) | Ulcerative colitis | -0.001692 |
| PGS002085(portability-ldpred2_702.2) | Seborrheic keratosis | 0.001679 |
| PGS000276(PlGF) | Placenta growth factor (PlGF) serum levels | 0.001675 |
| PGS001658(GBE_INI25509) | WA FA in tract superior longitudinal fasciculus (L) | -0.001672 |
| PGS001172(GBE_INI30150) | Eosinophill count | -0.001655 |
| PGS001592(GBE_INI25842) | Volume of grey matter in Precuneous Cortex (L) | -0.001649 |
| PGS000640(PRSWEB_PHECODE201_UKBB-SAIGE-HRC-X201_LASSOSUM_MGI_20200608) | Hodgkin's disease | 0.001644 |
| PGS000924(GBE_HC702) | Disorders of porphyrin and bilirubin metabolism (time-to-event) | 0.001642 |
| PGS000687(snpnet.IGF_1) | IGF-1 [nmol/L] | 0.001625 |
| PGS000479(PRSWEB_PHECODE174.1_C3-BREAST-3_LASSOSUM_MGI_20200608) | Breast cancer (female) | -0.001594 |
| PGS001008(GBE_QT_FC1002178) | Overall health rating | -0.001582 |
| PGS002689(disease_ALLERGY_ECZEMA_DIAGNOSED.SBayesR) | Eczema | -0.001572 |
| PGS000728(CKD_PGS) | Chronic kidney disease | -0.001568 |
| PGS000781(GRS7_Glio) | Glioma | -0.001557 |
| PGS000284(ST2) | ST2 protein (ST2) serum levels | -0.001530 |
| PGS001391(GBE_INI20453) | Ever taken cannabis | 0.001525 |
| PGS002161(portability-ldpred2_log_BMI) | Body mass index (BMI) | 0.001503 |
| PGS000301(GRS970_SBP) | Systolic blood pressure | -0.001500 |
| PGS001580(GBE_INI25860) | Volume of grey matter in Occipital Fusiform Gyrus (L) | -0.001489 |
| PGS001129(GBE_BIN_FC30022506) | Smoking status (ever vs never smokers) | 0.001483 |
| PGS000311(GRS234_TC) | Total cholesterol | 0.001475 |
| PGS002231(portability-ldpred2_years_of_edu) | Qualifications (years of education) | -0.001475 |
| PGS001367(GBE_INI5097) | 6mm weak meridian (L) | 0.001469 |
| PGS002114(portability-ldpred2_diastolic_BP) | Diastolic blood pressure, automated reading | 0.001459 |
| PGS000608(PRSWEB_PHECODE189.2_C3-BLADDER_PRS-CS_MGI_20200608) | Cancer of bladder | -0.001433 |
| PGS000799(GRSw_TAGC) | Asthma | 0.001430 |
| PGS002368(body_HEIGHTz.BOLT-LMM-BBJ) | Height | 0.001430 |
| PGS000299(GRS462_WHRadjBMI) | Waist-to-hip ratio (body mass index adjusted) | 0.001421 |
| PGS002257(GRS901_SBP) | Systolic blood pressure | -0.001420 |
| PGS002292(PRS36_KC) | Keratoconus | -0.001418 |
| PGS002222(portability-ldpred2_self_harm_thoughts) | Ever contemplated self-harm / Recent thoughts of suicide or self-harm | -0.001397 |
| PGS002323(disease_ALLERGY_ECZEMA_DIAGNOSED.BOLT-LMM) | Eczema | -0.001387 |
| PGS002104(portability-ldpred2_bad_hearing) | Hearing difficulty/problems | -0.001370 |
| PGS001903(portability-PLR_ECG_PP_interval) | PP interval | -0.001367 |
| PGS001003(GBE_INI137) | Number of medications taken | -0.001345 |
| PGS000961(GBE_HC987) | Phlebitis and thrombophlebitis (time-to-event) | 0.001318 |
| PGS001140(GBE_HC1190) | Seborrheic keratosis (time-to-event) | 0.001313 |
| PGS000222(CCL4) | C-C motif chemokine 4 (CCL4) serum levels | -0.001308 |
| PGS001995(portability-PLR_narcolepsy) | Daytime dozing / sleeping (narcolepsy) | -0.001301 |
| PGS001588(GBE_INI25869) | Volume of grey matter in Planum Polare (R) | 0.001294 |
| PGS001563(GBE_INI25886) | Volume of grey matter in Hippocampus (L) | -0.001289 |
| PGS000242(Gal-3) | Galectin-3 (Gal-3) serum levels | -0.001278 |
| PGS001526(GBE_INI22331) | QT interval | 0.001275 |
| PGS002105(portability-ldpred2_birth_weight) | Birth weight | 0.001271 |
| PGS002197(portability-ldpred2_log_triglycerides) | Triglycerides | 0.001260 |
| PGS002178(portability-ldpred2_log_IGF1) | IGF-1 | 0.001252 |
| PGS001049(GBE_BIN2040) | Risk taking behaviour | -0.001248 |
| PGS002691(blood_EOSINOPHIL_COUNT.SBayesR) | Eosinophil count | -0.001237 |
| PGS001864(portability-PLR_594) | Urinary calculus | 0.001234 |
| PGS002293(PRS62_psoriasis) | Psoriasis | -0.001224 |
| PGS001557(GBE_INI25862) | Volume of grey matter in Frontal Operculum Cortex (L) | -0.001200 |
| PGS001581(GBE_INI25877) | Volume of grey matter in Occipital Pole (R) | 0.001174 |
| PGS002031(portability-ldpred2_275.1) | Disorders of iron metabolism | 0.001170 |
| PGS001935(portability-PLR_less_happy_with_health) | General happiness with own health | -0.001161 |
| PGS002745 (metaPGS_RA) | Rheumatoid arthritis | -0.001159 |
| PGS000842(WHR) | Waist-hip ratio | 0.001150 |
| PGS001473(GBE_INI25370) | Mean ICVF in posterior corona radiata on FA skeleton (R) | -0.001136 |
| PGS001728(GBE_INI25622) | WA L2 in tract uncinate fasciculus (R) | 0.001134 |
| PGS000054(ALZ21_EFIGA) | Alzheimer's disease (late onset) | 0.001111 |
| PGS001273(GBE_HC22) | Osteoporosis | 0.001106 |
| PGS001099(GBE_INI5085) | Spherical power (left eye) | 0.001104 |
| PGS002281(PRS23_MM) | Multiple myeloma | -0.001101 |
| PGS000604(PRSWEB_PHECODE187.2_GWAS-Catalog-r2019-05-03-X187.2_PT_UKB_20200608) | Malignant neoplasm of testis | 0.001094 |
| PGS001779(BRSprs) | Brugada syndrome | 0.001093 |
| PGS000821(PRS_hypomed) | Thyroid medication use | -0.001090 |
| PGS002359(disease_AID_ALL.BOLT-LMM-BBJ) | Autoimmune disease | 0.001085 |
| PGS000685(snpnet.Glycated_haemoglobin_HbA1c) | HbA1c [mmol/mol] | 0.001067 |
| PGS001996(portability-PLR_neuroticism) | Neuroticism score | 0.001050 |
| PGS002767 (Knee_osteoarthritis_prscs) | Knee osteoarthritis | -0.001032 |
| PGS001128(GBE_BIN_FC20020116) | Previous Smoker | -0.001032 |
| PGS002314(body_BALDING1.BOLT-LMM) | Balding Type 1 | 0.001032 |
| PGS001047(GBE_BIN_FC20001249) | Past tobacco smoking (Smoked occasionally) | -0.001026 |
| PGS000817(GRS200_GGT) | Gamma-glutamyl transferase | -0.001021 |
| PGS001621(GBE_INI25897) | Volume of grey matter in VI Cerebellum (L) | -0.001020 |
| PGS001951(portability-PLR_log_fat_mass) | Whole body fat mass | 0.001008 |
| PGS001854(portability-PLR_550.1) | Inguinal hernia | -0.001006 |
| PGS001495(GBE_INI25443) | Mean ISOVF in body of corpus callosum on FA skeleton | 0.000996 |
| PGS001623(GBE_INI25906) | Volume of grey matter in VIIb Cerebellum (L) | 0.000975 |
| PGS001830(portability-PLR_318) | Tobacco use disorder | -0.000973 |
| PGS001842(portability-PLR_428) | Congestive heart failure; nonhypertensive | 0.000963 |
| PGS001717(GBE_INI25600) | WA L2 in tract cingulate gyrus part of cingulum (L) | 0.000943 |
| PGS001953(portability-PLR_log_HbA1c) | Glycated haemoglobin (HbA1c) | 0.000926 |
| PGS001593(GBE_INI25882) | Volume of grey matter in Putamen (L) | -0.000926 |
| PGS000140(GPpsy) | Broad Depression (seen a General Practitioner for nerves, anxiety, tension or depression) | -0.000908 |
| PGS001490(GBE_INI25391) | Mean ICVF in tapetum on FA skeleton (L) | -0.000895 |
| PGS001716(GBE_INI25599) | WA L2 in tract anterior thalamic radiation (R) | -0.000892 |
| PGS002342(mental_NEUROTICISM.BOLT-LMM) | Neuroticism | 0.000892 |
| PGS001607(GBE_INI25797) | Volume of grey matter in Temporal Pole (R) | -0.000888 |
| PGS000722(PRS_Kidney) | Kidney cancer | 0.000887 |
| PGS002318(other_MORNINGPERSON.BOLT-LMM) | Chronotype (morning person) | -0.000886 |
| PGS000010(GRS27) | Coronary heart disease | -0.000885 |
| PGS001834(portability-PLR_362.29) | Macular degeneration (senile) of retina NOS | -0.000876 |
| PGS001694(GBE_INI25717) | WA ISOVF in tract inferior fronto-occipital fasciculus (R) | -0.000876 |
| PGS000776(GRS9_Cirr) | Cirrhosis | 0.000866 |
| PGS000772(GRS95_SLEgen) | Systemic lupus erythematosus | -0.000864 |
| PGS001583(GBE_INI25848) | Volume of grey matter in Parahippocampal Gyrus, anterior division (L) | -0.000861 |
| PGS001288(GBE_HC95) | Inflammatory bowel disease | -0.000843 |
| PGS002065(portability-ldpred2_550.1) | Inguinal hernia | -0.000840 |
| PGS002145(portability-ldpred2_headaches_for_3m) | Headaches for 3+ months | -0.000831 |
| PGS001061(GBE_INI1289) | Cooked vegetable consumption | -0.000831 |
| PGS001713(GBE_INI25594) | WA L1 in tract uncinate fasciculus (L) | 0.000831 |
| PGS001687(GBE_INI25676) | WA ICVF in tract uncinate fasciculus (R) | -0.000827 |
| PGS000237(FABP4) | Fatty acid-binding protein, adipocyte (FABP4) serum levels | 0.000827 |
| PGS000993(GBE_QT_FC1001329) | Oily fish consumption | -0.000817 |
| PGS001795(1kgeur_gbmi_leaveUKBBout_UtC_pst_eff_a1_b0.5_phiauto) | Uterine cancer | -0.000817 |
| PGS002295(GRS413_IGF-1) | Insulin growth-like factor-1 level | 0.000794 |
| PGS000281(RETN) | Resistin (RETN) serum levels | 0.000789 |
| PGS000856(T2D_LiverLipids) | Type 2 diabetes (based on SNPs associated with liver lipids) | 0.000786 |
| PGS000651(PRSWEB_PHECODE204.12_UKBB-SAIGE-HRC-X204.12_LASSOSUM_MGI_20200608) | Lymphoid leukemia, chronic | -0.000783 |
| PGS000329(PRS_CHD) | Coronary heart disease | -0.000761 |
| PGS001910(portability-PLR_ever_cannabis) | Ever taken cannabis | -0.000748 |
| PGS001337(GBE_FH1002) | Family history of breast cancer | -0.000747 |
| PGS001046(GBE_BIN_FC40001249) | Past tobacco smoking (Smoked at least once) | -0.000746 |
| PGS001810(portability-PLR_200.1) | Polycythemia vera | 0.000744 |
| PGS002288(PRS_POP) | Pelvic organ prolapse | 0.000743 |
| PGS000891(GLGC_2021_EAS_LDL_PRS_weights_PT) | Low density lipoprotein (LDL) cholesterol | 0.000740 |
| PGS001610(GBE_INI25895) | Volume of grey matter in V Cerebellum (L) | -0.000713 |
| PGS001068(GBE_INI1548) | Variation in diet | -0.000709 |
| PGS001544(GBE_INI25006) | Volume of grey matter | -0.000697 |
| PGS000871(IS_14) | Insulin secretion | -0.000696 |
| PGS002174(portability-ldpred2_log_heel_BUA) | Heel Broadband ultrasound attenuation, direct entry | -0.000694 |
| PGS001400(GBE_INI30020) | Haemoglobin concentration | -0.000689 |
| PGS000857(T2D_Obesity) | Type 2 diabetes (based on SNPs associated with obesity) | 0.000672 |
| PGS002311(disease_ASTHMA_DIAGNOSED.BOLT-LMM) | Asthma | -0.000670 |
| PGS002787(BD1_SDPR) | Type 1 bipolar disorder | -0.000662 |
| PGS000326(GRS-JIA-RFN-20) | Rheumatoid-factor-negative Polyarthritis (Juvenile Idiophatic Arthritis) | -0.000661 |
| PGS002352(biochemistry_Cholesterol.BOLT-LMM) | Total cholesterol | 0.000660 |
| PGS000230(CXCL1) | C-X-C motif chemokine 1 (CXCL1) serum levels | -0.000648 |
| PGS001484(GBE_INI25369) | Mean ICVF in superior corona radiata on FA skeleton (L) | -0.000636 |
| PGS001476(GBE_INI25373) | Mean ICVF in posterior thalamic radiation on FA skeleton (L) | -0.000630 |
| PGS002588(disease_DERMATOLOGY.P+T.5e-08) | Dermatologic diseases | -0.000623 |
| PGS001645(GBE_INI25490) | WA FA in tract anterior thalamic radiation (L) | 0.000604 |
| PGS000365(PRSWEB_PHECODE150_C3-OESOPHAGUS_PRS-CS_MGI_20200608) | Cancer of esophagus | -0.000601 |
| PGS001770(GBE_INI25699) | WA OD in tract superior longitudinal fasciculus (R) | 0.000598 |
| PGS001586(GBE_INI25851) | Volume of grey matter in Parahippocampal Gyrus, posterior division (R) | -0.000595 |
| PGS001078(GBE_INI30190) | Monocyte % | -0.000593 |
| PGS001916(portability-PLR_fall_1y) | Falls in the last year | 0.000590 |
| PGS000908(PRS_Insomnia) | Insomnia | -0.000590 |
| PGS001616(GBE_INI25898) | Volume of grey matter in Vermis VI Cerebellum | 0.000585 |
| PGS001005(GBE_INI134) | Number of self reported cancers | 0.000583 |
| PGS001514(GBE_INI87) | Non-cancer illness year/age first occurred | 0.000563 |
| PGS002283(GRS15_NAFLD) | Nonalcoholic fatty liver disease | -0.000545 |
| PGS000718(PRPBB_44) | Beta-blocker survival benefit | 0.000538 |
| PGS000780(PRS135_allergy) | Allergic diseases | -0.000535 |
| PGS002017(portability-ldpred2_189.2) | Cancer of bladder | -0.000530 |
| PGS002125(portability-ldpred2_ever_cannabis) | Ever taken cannabis | -0.000526 |
| PGS000139(MDDRecur) | Lifetime Major Depressive Disorder (with recurrence) | -0.000519 |
| PGS000249(IL-18) | Interleukin-18 (IL-18) serum levels | -0.000513 |
| PGS001756(GBE_INI25539) | WA MD in tract superior thalamic radiation (R) | 0.000508 |
| PGS000858(T2D_Proinsulin) | Type 2 diabetes (based on SNPs associated with proinsulin levels) | 0.000506 |
| PGS002020(portability-ldpred2_211) | Benign neoplasm of other parts of digestive system | 0.000501 |
| PGS001513(GBE_INI25027) | Median T2star in thalamus (R) | -0.000497 |
| PGS000292(TRANCE) | TNF-related activation-induced cytokine (TRANCE) serum levels | 0.000493 |
| PGS002678(disease_AID_ALL.SBayesR) | Autoimmune disease | -0.000488 |
| PGS000628(PRSWEB_PHECODE193_C3-THYROID-GLAND_LASSOSUM_MGI_20200608) | Thyroid cancer | -0.000488 |
| PGS000217(ADM) | Adrenomedullin (ADM) serum levels | 0.000488 |
| PGS000324(GRS-JIA-ERA-20) | Enthesitis-related Juvenile Idiophatic Arthritis | -0.000481 |
| PGS001114(GBE_BIN_FC8006154) | Ibuprofen use self-reported | -0.000474 |
| PGS000303(GRS253_eGFR) | Estimated glomerular filtration rate | -0.000466 |
| PGS002218(portability-ldpred2_poorer_health) | Overall health rating | 0.000465 |
| PGS001292(GBE_FH1044) | Family history of prostate cancer | 0.000462 |
| PGS002316(disease_CARDIOVASCULAR.BOLT-LMM) | Cardiovascular disease | 0.000457 |
| PGS001603(GBE_INI25854) | Volume of grey matter in Temporal Fusiform Cortex, anterior division (L) | -0.000451 |
| PGS000634(PRSWEB_PHECODE193_UKBB-SAIGE-HRC-X193_PRS-CS_MGI_20200608) | Thyroid cancer | -0.000440 |
| PGS001814(portability-PLR_241.2) | Nontoxic multinodular goiter | 0.000440 |
| PGS000258(KLK6) | Kallikrein-6 (KLK6) serum levels | -0.000437 |
| PGS000080(CC_NHL) | Non-Hodgkin's lymphoma | 0.000437 |
| PGS002074(portability-ldpred2_593) | Hematuria | 0.000423 |
| PGS000387(PRSWEB_PHECODE165_UKBB-SAIGE-HRC-X165_LASSOSUM_MGI_20200608) | Cancer within the respiratory system | -0.000420 |
| PGS000234(ECP) | Eosinophil cationic protein (ECP) serum levels | 0.000406 |
| PGS001550(GBE_INI25838) | Volume of grey matter in Cingulate Gyrus, anterior division (L) | 0.000391 |
| PGS001611(GBE_INI25896) | Volume of grey matter in V Cerebellum (R) | 0.000389 |
| PGS001351(MAGICTA_EUR_PGS_FI) | Fasting insulin | 0.000382 |
| PGS001375(GBE_INI22426) | Average heart rate | -0.000374 |
| PGS002785(SCZ_SDPR) | Schizophrenia | 0.000372 |
| PGS002677(disease_ASTHMA_DIAGNOSED.SBayesR) | Asthma | -0.000364 |
| PGS000232(CXCL6) | C-X-C motif chemokine 6 (CXCL6) serum levels | -0.000361 |
| PGS001735(GBE_INI25635) | WA L3 in tract inferior fronto-occipital fasciculus (L) | 0.000360 |
| PGS000087(CC_Thyroid) | Thyroid cancer | 0.000351 |
| PGS000654(PRSWEB_PHECODE204.4_GWAS-Catalog-r2019-05-03-X204.4_PT_UKB_20200608) | Multiple myeloma | -0.000346 |
| PGS001494(GBE_INI25462) | Mean ISOVF in anterior corona radiata on FA skeleton (R) | -0.000343 |
| PGS001829(portability-PLR_296.2) | Depression | 0.000342 |
| PGS000077(CC_LL) | Lymphocytic leukemia | -0.000337 |
| PGS000944(GBE_HC261) | Eczema, dermatitis | -0.000303 |
| PGS001251(GBE_HC1052) | Other interstitial pulmonary diseases (time-to-event) | 0.000299 |
| PGS002738(PRS_AUD) | Alcohol use disorder | 0.000295 |
| PGS001333(GBE_HC1582) | Chronic obstructive pulmonary disease (algorithmically-defined) | 0.000284 |
| PGS002198(portability-ldpred2_log_urea) | Urea | -0.000283 |
| PGS000730(PRS_BCC) | Basal cell carcinoma | 0.000282 |
| PGS001069(GBE_INI1528) | Water intake | -0.000278 |
| PGS001817(portability-PLR_250.1) | Type 1 diabetes | 0.000270 |
| PGS002043(portability-ldpred2_365) | Glaucoma | -0.000270 |
| PGS000126(Urate_GRS) | Serum urate | -0.000265 |
| PGS001767(GBE_INI25692) | WA OD in tract inferior longitudinal fasciculus (R) | 0.000260 |
| PGS001126(GBE_INI1498) | Coffee intake | 0.000256 |
| PGS001062(GBE_INI1309) | Fresh fruit intake | -0.000256 |
| PGS001882(portability-PLR_740) | Osteoarthrosis | -0.000244 |
| PGS002156(portability-ldpred2_log_age_first_sex) | Age first had sexual intercourse | -0.000243 |
| PGS000747(PRS_EB) | Coronary artery disease | 0.000232 |
| PGS002680(body_BALDING1.SBayesR) | Balding Type 1 | 0.000231 |
| PGS001983(portability-PLR_log_waist_circ) | Waist circumference | 0.000219 |
| PGS001791(1kgeur_gbmi_leaveUKBBout_IPF_pst_eff_a1_b0.5_phiauto) | Idiopathic pulmonary fibrosis | 0.000217 |
| PGS001868(portability-PLR_654.2) | Rhesus isoimmunization in pregnancy | 0.000213 |
| PGS001419(GBE_INI25091) | Mean FA in cingulum cingulate gyrus on FA skeleton (L) | -0.000209 |
| PGS002284(GRS_286_HDL) | High density lipoprotein cholesterol | 0.000177 |
| PGS000625(PRSWEB_PHECODE191.11_UKBB-SAIGE-HRC-X191.11_PT_MGI_20200608) | Cancer of brain | 0.000170 |
| PGS002370(blood_LYMPHOCYTE_COUNT.BOLT-LMM-BBJ) | Lymphocyte Count | 0.000168 |
| PGS001502(GBE_INI25471) | Mean ISOVF in sagittal stratum on FA skeleton (L) | 0.000161 |
| PGS001252(GBE_BIN_FC3002247) | Hearing difficulty and deafness | -0.000160 |
| PGS000305(GRS31_FG) | Fasting glucose | -0.000155 |
| PGS002122(portability-ldpred2_erythrocyte_width) | Red blood cell (erythrocyte) distribution width | -0.000135 |
| PGS002362(bp_DIASTOLICadjMEDz.BOLT-LMM-BBJ) | Diastolic blood pressure | -0.000121 |
| PGS002763 (Hip_osteoarthritis_prscs) | Hip osteoarthritis | -0.000120 |
| PGS001656(GBE_INI25507) | WA FA in tract posterior thalamic radiation (L) | 0.000114 |
| PGS001331(GBE_HC322) | Crohns disease | 0.000114 |
| PGS002346(blood_RBC_DISTRIB_WIDTH.BOLT-LMM) | Red Blood Cell Distribution Width | -0.000110 |
| PGS000255(IL16) | Pro-interleukin-16 (IL16) serum levels | -0.000109 |
| PGS000338(GRS97_AF) | Atrial fibrillation | 0.000104 |
| PGS002298(PRS14_esophageal) | Esophageal cancer | 0.000104 |
| PGS001759(GBE_INI25553) | WA MO in tract forceps minor | -0.000103 |
| PGS000096(irf) | Immature fraction of reticulocytes | 0.000094 |
| PGS001055(GBE_QT_FC1001180) | Chronotype (morning/evening person) | 0.000078 |
| PGS002121(portability-ldpred2_ECG_RR_interval) | RR interval | -0.000074 |
| PGS002728(PRS_hip) | Hip osteoarthritis | 0.000067 |
| PGS001418(GBE_INI25071) | Mean FA in cerebral peduncle on FA skeleton (L) | -0.000062 |
| PGS001226(GBE_INI20022) | Birth weight | 0.000058 |
| PGS001538(GBE_INI25024) | Volume of accumbens (R) | 0.000054 |
| PGS001264(GBE_HC166) | Deep vein thrombosis | 0.000050 |
| PGS001579(GBE_INI25805) | Volume of grey matter in Middle Temporal Gyrus, posterior division (R) | -0.000042 |
| PGS000357(PRSWEB_PHECODE145_C3-LIP-ORAL-PHARYNX_PT_MGI_20200608) | Cancer of mouth | 0.000032 |
| PGS000446(PRSWEB_PHECODE172.21_20001-1061_PRS-CS_MGI_20200608) | Basal cell carcinoma | 0.000032 |
| PGS001771(GBE_INI25701) | WA OD in tract superior thalamic radiation (R) | 0.000024 |
| PGS002217(portability-ldpred2_play_computer) | Plays computer games | -0.000024 |
| PGS002140(portability-ldpred2_glasses) | Wears glasses or contact lenses | -0.000021 |
| PGS002193(portability-ldpred2_log_pulse_rate) | Pulse rate, automated reading | 0.000017 |
| PGS001629(GBE_INI25920) | Volume of grey matter in X Cerebellum (R) | 0.000016 |
| PGS001259(GBE_HC49) | Hayfever/allergic rhinitis | -0.000007 |
| PGS000937(GBE_HC401) | Varicose veins | 0.000003 |
| PGS001917(portability-PLR_fat_perc) | Body fat percentage | 0.000003 |

# Table S3. Sex stratified analysis (Female): Odds Ratio (OR) estimates, 95% CI and p-values for each predictor in logistic regression model

| Model No. | Predictors | OR Estimates | 95% CI: Lower limit | 95% CI: Upper limit | P-value |
| --- | --- | --- | --- | --- | --- |
| 1 | CRC-KL200 | 1.42 | 1.34 | 1.51 | <0.0001 |
| 2 | CRC-LDpred | 1.60 | 1.51 | 1.70 | <0.0001 |
| 3 | CRC-KL200 | 1.08 | 1 | 1.16 | 0.057 |
|  | CRC-LDpred | 1.52 | 1.40 | 1.65 | <0.0001 |
| 4 | Composite MPS | 1.25 | 1.17 | 1.34 | <0.0001 |
|  | CRC-KL200 | 1.27 | 1.19 | 1.36 | <0.0001 |
| 5 | Composite MPS | 1.14 | 1.07 | 1.22 | 0.00013 |
|  | CRC-LDpred | 1.49 | 1.39 | 1.60 | <0.0001 |
| 6 | Composite MPS | 1.13 | 1.06 | 1.21 | 0.0005 |
|  | CRC-KL200 | 1.05 | 0.97 | 1.14 | 0.26 |
|  | CRC-LDpred | 1.45 | 1.34 | 1.57 | <0.0001 |

Abbreviations. CRC: colorectal cancer. CI: confidence interval. MPS: multiple PRS.

# Table S4. Sex stratified analysis (Male): Odds Ratio (OR) estimates, 95% CI and p-values for each predictor in logistic regression model

| Model No. | Predictors | OR estimates | 95% CI: Lower limit | 95% CI: Upper limit | P-value |
| --- | --- | --- | --- | --- | --- |
| 1 | CRC-KL200 | 1.52 | 1.45 | 1.62 | <0.0001 |
| 2 | CRC-LDpred | 1.65 | 1.55 | 1.75 | <0.0001 |
| 3 | CRC-KL200 | 1.19 | 1.09 | 1.28 | <0.0001 |
|  | CRC-LDpred | 1.48 | 1.36 | 1.58 | <0.0001 |
| 4 | Composite MPS | 1.23 | 1.15 | 1.31 | <0.0001 |
|  | CRC-KL200 | 1.39 | 1.30 | 1.48 | <0.0001 |
| 5 | Composite MPS | 1.15 | 1.07 | 1.23 | <0.0001 |
|  | CRC-LDpred | 1.52 | 1.43 | 1.63 | <0.0001 |
| 6 | Composite MPS | 1.12 | 1.05 | 1.20 | 0.0011 |
|  | CRC-KL200 | 1.16 | 1.07 | 1.25 | 0.0003 |
|  | CRC-LDpred | 1.40 | 1.30 | 1.52 | <0.0001 |

Abbreviations. CRC: colorectal cancer. CI: confidence interval. MPS: multiple PRS.

# Table S5. CRC analysis: Odds Ratio (OR) estimates, 95% CI and p-values for each predictor in logistic regression model

| Model No. | Predictors | OR Estimates | 95% CI: Lower limit | 95% CI: Upper limit | P-value |
| --- | --- | --- | --- | --- | --- |
| 1 | CRC-KL200 | 1.57 | 1.45 | 1.70 | <0.0001 |
| 2 | CRC-LDpred | 1.79 | 1.65 | 1.93 | <0.0001 |
| 3 | CRC-KL200 | 1.13 | 1.02 | 1.26 | 0.021 |
|  | CRC-LDpred | 1.65 | 1.49 | 1.84 | <0.0001 |
| 4 | Composite MPS | 1.22 | 1.12 | 1.34 | <0.0001 |
|  | CRC-KL200 | 1.43 | 1.31 | 1.57 | <0.0001 |
| 5 | Composite MPS | 1.09 | 1 | 1.20 | 0.059 |
|  | CRC-LDpred | 1.72 | 1.55 | 1.88 | <0.0001 |
| 6 | Composite MPS | 1.07 | 0.97 | 1.17 | 0.15 |
|  | CRC-KL200 | 1.12 | 1 | 1.25 | 0.051 |
|  | CRC-LDpred | 1.62 | 1.43 | 1.80 | <0.0001 |

Abbreviations. CRC: colorectal cancer. CI: confidence interval. MPS: multiple PRS.

# Table S6. Sex stratified analysis Females: Comparison of AUCs 95% CI and p-values

| Comparison pair No. | Model A | Model B | Point estimate of difference in AUC | 95% CI of the difference in AUC | P-value |
| --- | --- | --- | --- | --- | --- |
| 1 | CRC-KL200 + MPS | CRC-KL200 | 0.012 | 0.003, 0.021 | 0.008 |
| 2 | CRC-LDpred + MPS | CRC-LDpred | 0.004 | 0.001, 0.009 | 0.031 |
| 3 | CRC-KL200 + CRC-LDpred + MPS | CRC-KL200 + CRC-LDpred | 0.003 | 0.000, 0.007 | 0.067 |

Abbreviations. AUC: area under the Receiver Operating Curve. CRC: colorectal cancer. CI: confidence interval. MPS: multiple PRS.

# Table S7. Sex Stratified analysis Males: Comparison of AUCs,

# 95% CI and p-values

#

| Comparison pair No. | Model A | Model B | Point estimate of difference in AUC | 95% CI of the difference in AUC | P-value |
| --- | --- | --- | --- | --- | --- |
| 1 | CRC-KL200 + MPS | CRC-KL200 | 0.016 | 0.009, 0.023 | <0.0001 |
| 2 | CRC-LDpred + MPS | CRC-LDpred | 0.005 | 0.001, 0.010 | 0.015 |
| 3 | CRC-KL200 + CRC-LDpred + MPS | CRC-KL200 + CRC-LDpred | 0.004 | 0.001, 0.008 | 0.019 |

Abbreviations. AUC: area under the Receiver Operating Curve. CRC: colorectal cancer. CI: confidence interval. MPS: multiple PRS.

# Table S8. CRC analysis: Comparison of AUCs, 95% CI and p-values (CRC PRS-specific models with CRC PRS and MPS models)

| Comparison pair No. | Model A | Model B | Point estimate of difference in AUC | 95% CI of the difference in AUC | P-value |
| --- | --- | --- | --- | --- | --- |
| 1 | CRC-KL200 + MPS | CRC-KL200 | 0.015 | 0.007, 0.023 | 0.0002 |
| 2 | CRC-LDpred + MPS | CRC-LDpred | 0.002 | 0.000, 0.015 | 0.13 |
| 3 | CRC-KL200 + CRC-LDpred + MPS | CRC-KL200 + CRC-LDpred | 0.002 | 0.000, 0.004 | 0.098 |

Abbreviations. AUC: area under the Receiver Operating Curve. CRC: colorectal cancer. CI: confidence interval. MPS: multiple PRS.


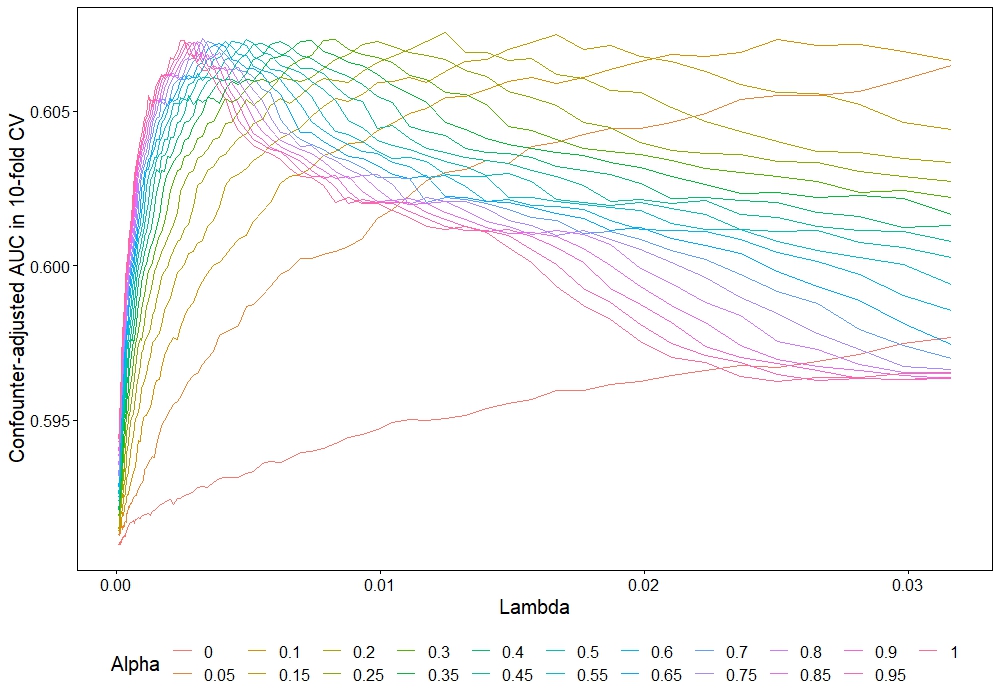


# Figure S1. Confounder-adjusted AUC in 10-fold CV

The mean value of the confounder-adjusted AUC in 10-fold CV by different combinations of alpha and lambda values in Elastic Net models.

Abbreviations. AUC: area under the Receiver Operating Curve. CV: cross-validation. ML: machine learning.


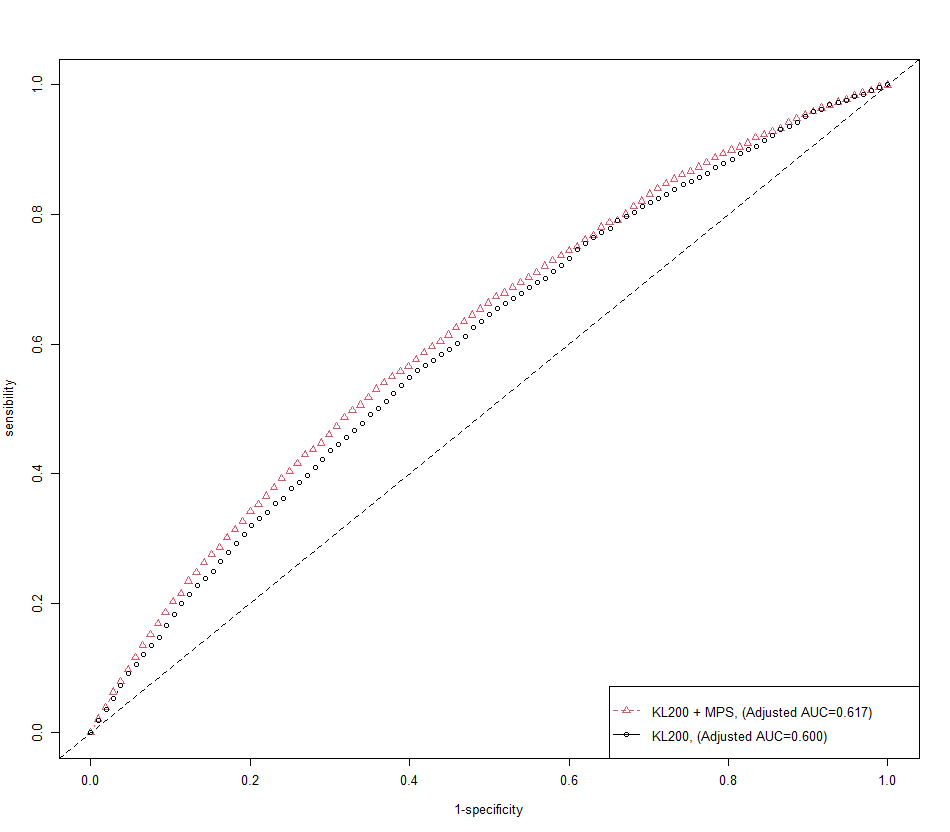


# Figure S2. Main analysis: Confounder-adjusted AUC comparing models MPS+CRC-KL200 vs. CRC-KL200 only

The red line is the confounder-adjusted ROC curve of a risk prediction model with CRC-KL200 and MPS, and the AUC was 0.617 in the validation dataset; the black line is the confounder-adjusted ROC curve of a risk prediction model with CRC-KL200 only, and the AUC was 0.600 in the validation dataset.

Abbreviations. AUC: area under the Receiver Operating Curve. CRC: colorectal cancer. CI: confidence interval. MPS: multiple PRS. ROC: Receiver Operating Curve.


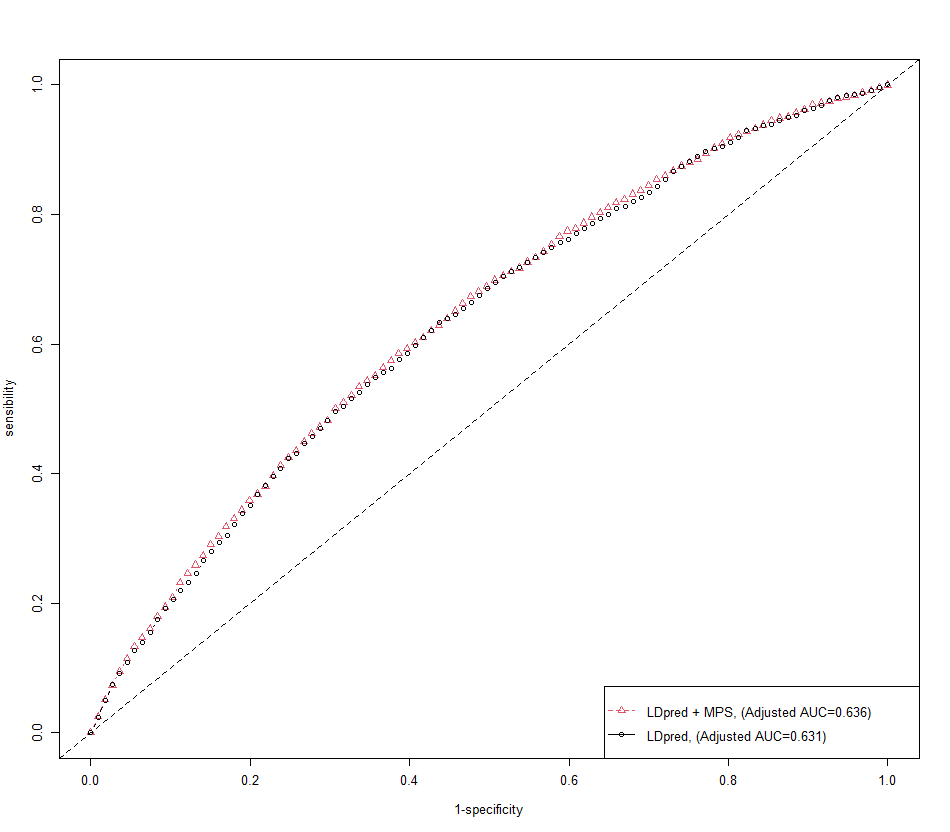


# Figure S3. Main analysis: Confounder-adjusted AUC comparing models MPS+CRC-LDpred vs. CRC-LDpred only

The red line is the confounder-adjusted ROC curve of a risk prediction model with CRC-LDpred and MPS, and the AUC was 0.636 in the validation dataset; the black line is the confounder-adjusted ROC curve of a risk prediction model with CRC-LDpred only, and the AUC was 0.631 in the validation dataset.

Abbreviations. AUC: area under the Receiver Operating Curve. CRC: colorectal cancer. CI: confidence interval. MPS: multiple PRS. ROC: Receiver Operating Curve.


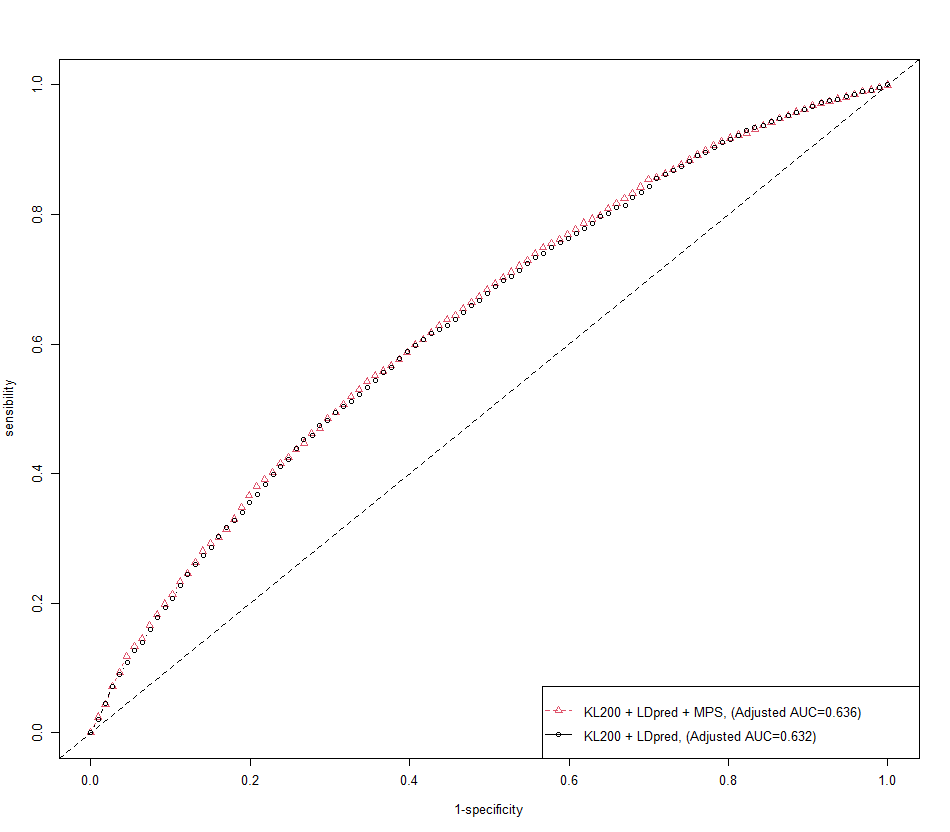


# Figure S4. Main analysis: Confounder-adjusted AUC comparing models MPS+CRC-KL200+CRC-LDpred vs. CRC-KL200+CRC-LDpred

The red line is the confounder-adjusted ROC curve of a risk prediction model with CRC-KL200, CRC-LDpred, and MPS, and the AUC was 0.636 in the validation dataset; the black line is the confounder-adjusted ROC curve of a risk prediction model with CRC-KL200 combined with CRC-LDpred, and the AUC was 0.632 in the validation dataset.

Abbreviations. AUC: area under the Receiver Operating Curve. CRC: colorectal cancer. CI: confidence interval. MPS: multiple PRS. ROC: Receiver Operating Curve.
